# Retinoic acid alters metalloproteinase action in red deer antler stem cells

**DOI:** 10.1371/journal.pone.0287782

**Published:** 2023-07-10

**Authors:** Anna J. Korzekwa, Anna Kononiuk, Władysław Kordan, Aleksandra Orzołek

**Affiliations:** 1 Department of Biodiversity Protection (DBP), Institute of Animal Reproduction and Food Research, Polish Academy of Sciences (IAR&FR PAS), Olsztyn, Poland; 2 Department of Animal Biochemistry and Biotechnology, Faculty of Animal Bioengineering, University of Warmia and Mazury in Olsztyn, Olsztyn, Poland; Universidade de Trás-os-Montes e Alto Douro: Universidade de Tras-os-Montes e Alto Douro, PORTUGAL

## Abstract

Metalloproteinases (MMP)s regulate developmental processes, control angiogenesis and wound healing, participate in the formation of immune receptors, and are expressed in stem cells. Retinoic acid (RA) is a potential modulator of these proteinases. The aim was to determine (1) MMPs’ action in antler stem cells (ASCs) before and after differentiation into adipo-, osteo-, and chondrocytes and (2) the effect of RA on modifying MMP action in ASCs. Antler tissue from pedicle was collected approximately 40 days after antler casting, *post mortem* from healthy breeding five year old males (N = 7). The cells were isolated from the pedicle layer of periosteum after skin separation and cultured. The pluripotency of the ASCs was evaluated by mRNA expression for NANOG, SOX2, and OCT4. ASCs were stimulated with RA (100nM) and differentiated for 14 days. The MMP (1–3) and TIMP(1–3) (tissue inhibitor of MMPs) mRNA expression was determined in the ASCs, their concentrations in the ASCs and the medium after RA stimulation as well as profiles of mRNA expression for MMPs: 1–3 and TIMPs: 1–3 during differentiation of ASC to osteocytes, adipocytes and chondrocytes. RA increased MMP-3 and TIMP-3 mRNA expression and output (P < 0.05) and not influenced on MMP-1 and TIMP-1 mRNA expression and output in ASC (P > 0.05). Depending on differentiation of ASC to osteocytes, adipocytes or chondrocytes, MMPs`and TIMPs`expression profile fluctuates for all studied proteases and its inhibitors. The studies demand continuation considering the role of proteases in stem cells physiology and differentiation. The results may be relevant for the study of cellular processes during the cancerogenesis of tumor stem cells.

## Introduction

The growth and annual regeneration of deer antlers are considered a phenomenon of regenerative processes in mammals. Every year after the antler casting, it self-renews by producing several tissues such as skin, blood, bone, and hair [1]. Antler regrowth does not occur in animals deprived of the pedicle. The stem cells responsible for the ability to regenerate are located in the pedicle [2, 3]. Antler stem cells (ASC) show the characteristics of both mesenchymal stem cells (expression of characteristic markers) and embryonic stem cells (ability to give rise to different types of tissues such as blood, skin, connective tissue, and bone) [4]. In addition, they are characterized by a significant renewal rate (antler growth approximately 1.7 cm per day) that surpasses even the growth of some cancerous tissues [1, 5]. Identical factors have been recognized in tumor cells and ASCs, such as heat shock protein 47 (HSP47) [6]. The morphogenesis and histogenesis of antler regeneration have been studied in detail [7, 8]. Recent studies have shown that deer antler velvet extract possesses antitumor activity [9] and expresses tumor suppressor genes [10]. Nevertheless, the cellular mechanism of antler-sourced factors on tumor inhibition is not yet known. However, undoubtedly one of the known factors affecting antler growth process is retinoic acid (RA), a derivative of vitamin A [11].

Vitamin A (retinol) and its natural and synthetic derivatives (retinoids) are involved in a wide spectrum of physiological processes such as reproduction, cell proliferation, differentiation, vision, and embryonic development [12, 13]. Moreover, several cancers are sensitive to retinoids, including epithelial cancers, precancerous lesions, and leukemia [14, 15]. On the other hand, increased intracellular metabolism during cancerogenesis has also been linked to cellular resistance to retinol analogues’ treatment, suggesting that some metabolites participate in retinoid signaling [16]. The retinoid signal is transduced by two families of nuclear receptors, the retinoic acid receptor (RAR) and the retinoid X receptor (RXR) family. Retinol in the cell is metabolized at first to retinal and then to RA [17]. The increase in antlerogenic periosteal cells’ proliferation *in vivo* after RA injection has been reported [18, 19].

The interaction between RA and metalloproteinase (MMP) action is possible taking into consideration its multiple roles. Metalloproteinases degrade the extracellular matrix components such as laminin, collagen, fibronectin, and proteoglycans. This facilitates cell migration and the regulation of the activity of growth factors. Additionally, MMPs are zinc-dependent and act on a variety of extracellular protein substrates, often to activate latent forms of effector proteins, including antimicrobial peptides and cytokines, or to alter protein function, such as the shedding of cell-surface proteins [20]. Because their substrates are diverse, MMPs are involved in a variety of homeostatic functions, such as bone remodeling, wound healing, developmental processes, control angiogenesis, and several aspects of immunity [21]. In the process of migration, plasticity, self-renewal, and the pluripotency of mesenchymal stem cells, a balance between the MMPs and TIMPs is maintained [22]. Matrix metalloproteinases are also involved in pathological processes. In the case of cancer, MMPs play a key role in the process of metastasis, due to their ability to allow cells to migrate by destroying the extracellular matrix barrier [23] and by enhancing angiogenesis [24], especially MMP-2 (initial stage of tumor progression and MMP-9 –invasive stage. Among more than 25 MMPs described, for our study we selected three basic ones, the best characterized in terms of action, but belonging to three different subgroups differing in quaternary structure and substrate specificity: MMP-1, a proteinase from the collagenase subgroup, MMP-2, a gelatinase, and MMP-3, a stromelysin. The function of MMPs is regulated by TIMP proteins, which have the property of limiting or blocking the activity of MMPs and are important regulators of the extracellular matrix turnover, tissue remodeling and cellular behavior. TIMPs have various biological activities such as promoting cell proliferation, anti-angiogenic, pro- and anti-apoptotic and synaptic plasticity activities, although many of TIMPs may act independently of MMP inhibition (e.g. TIMP3) [25].

The cartilage degradation model *in vitro* was characterized by aggrecan loss, but no collagen catabolism was stimulated with RA and exhibited further degeneration [26]. In contrast, RA increased the migration capacity of the mesenchymal stem cells *in vitro* by stimulating the expression and activity of MMP-2/-9 [27]. Stem cells are considered as a therapy in tendon transplants or dermatological conditions, and treatments have already been performed on animals [28, 29] and humans [30]. Therefore, we used deer antler cells as a model to study the interaction between RA and MMPs’ action in stem cells.

Antler stem cells are a promising basis for therapy, and RA participates in antler development, and also used as a "catalyst" therapy for cancer treatment. Metalloproteinases are involved in both remodeling, tissue regeneration and cell proliferation processes. With this in mind, we undertook our research on antlerogenic cells, which can differentiate into three cell types with RA involvement. Considering the intensive ossification processes that occur in red deer antlers, as well as the multiple actions of MMPs in extracellular matrix transformation, the aim of this study is to verify (1) the action of these proteases in ASCs before and after differentiation into adipo-, osteo-, and chondrocytes, and (2) the effect of RA on modifying MMPs`action in ASCs.

## Material and methods

### Sample collection

The experimental material (heads with antlers) was collected *post mortem* 15–20 min after the shot (15-20th May 2021) from healthy breeding five-year-old males red deer (*Cervus elaphus)* (farm in Elganowo; Northeast Poland; N = 7). The slaughter of the animals was the decision of the farm owner. The reasons for culling animals from the herd on the farm were economic considerations and herd renewal. Moreover, informed consent was obtained from the farm owner for the collection of samples. All the experiments were performed in accordance with the ARRIVE guidelines and regulations and were approved by the Welfare Ethics Committee in IAR&FR PAS. The heads were kept on ice until transport to the laboratory (max. 30 min.), and the tissues were prepared from the antlers immediately to isolate the ASCs.

### Primary antler cells’ isolation and culture

Tissue (pedicle periosteum) was collected from antler which was growth for approximately 40 days after casting from the pedicle after skin separation The cells were isolated and cultured separately from each individual. The collected antler tissue was minced with a surgical blade (Swann-Morton, Sheffield, UK) and scissors (Miltex, York, PA, USA) under sterile conditions. The digestion of the tissue separately from each individual was achieved by incubating with slow mixing for 30 min in Dulbecco Modified Eagle medium (DMEM; D-1152; Sigma, Darmstadt, Germany), containing 0.05% collagenase (C-0130; Sigma), 0.005% DNase I (D-5025; Sigma) and 0.1% (w/v) BSA (735078; Roche Diagnostics GmbH, Mannheim, Germany) in a water bath at 37°C; then, the mixture was centrifugated at 350 × g for 5 min. The cell viability was higher than 85% as assessed by trypan blue exclusion. The dispersed antler cells (2.0 × 10^5^/ml) were cultured in 100 μl of DMEM containing 5% CS (N4752; Sigma) and antibiotic–antimycotic solution (A5955, Sigma) in 6-well culture collagen-coated dishes (356400, Life Sciences; two plates were cultured from one individual). The cells were cultured in a humidified atmosphere containing 5% CO_2_ at 37,5°C. After achieving 50% confluency, the culture from the first plate was kept until 70% for stimulation with RA (experiment 1) and the culture from the second plate proceeded to differentiation (experiment 2). The medium was collected separately and kept at -20°C until the ELISA analyses. The ASCs in experiment 1 were rinsed with phosphate buffered saline (PBS, 10010001, Thermofisher, Wilmington, DE) sonicated, and lysed in 1 ml of lysis buffer (AR0103, Boster, Pleasanton, CA, US). Then, the lysates were centrifuged at approximately 10,000 x g and 4°C for 5 min. The supernatant was used for the ELISA analyses, whereas the cells for mRNA expression in the preliminary experiment and experiments 1 and 2. The negative control for the ASC culture was fibrocyte cell culture (tissue collected from the frontal part of the forehead after skin stripping) from each individual separately.

## Experimental procedure

### Preliminary experiments

#### Determination of pluripotent markers in mRNA expression in ASCs

The monolayer of the ASCs after achieving 70% confluency was examined for pluripotency potential. Briefly, the medium was removed from the dish, and 1 ml of TRI-Reagent (T9424, Sigma) was added to the cells per one dish. The cells were mechanically peeled off with the use of sterile scrapers (S5981, Sigma) and immediately frozen at -70°C. After RNA isolation and reverse transcription, the mRNA expression of the pluripotency markers, OCT4, NANOG, and SOX2, was determined by real-time PCR (the sequences of the primers are presented in Table 1).

**Table 1:** List of primer sequences used for real-time PCR.

Gene name	Primers sequence (5ʹ-3ʹ)	Amplicon length (bp)	EMBL
GAPDH	F: CACCCTCAAGATTGTCAGCA	103	BC102589
	R: GGTCATAAGTCCCTCCACGA		
MMP-1	F: GCTTTGGCTTCCCTAGCAGTG	201	NC_028491.1
	R: C TCGCCTTTTTGGAAAACATC		
MMP-2	F: TCTGCCCCCATGAAGCCCTGTT	347	NC_037345.1
	R: GCCCCACTTGCGGTCATCATCGTA		
MMP-3	F: AAGTTCCTTGGCTTGGAGGT	220	NC_037342.1
	R: ATCTCCATGTTCTCGGACTC		
TIMP-1	F: CATGGAGAGCCTCTGTGGAT	210	NC_037357.1
	R: ATGGCTGAACAGGGAAACAC		
TIMP-2	F: ATAGTGATCAGGGCCAAAGCAGTC	277	EF619492.1
	R: TGTCCCAGGGCACGATGAAGTC		
TIMP-3	F: GATGTACCGAGGATTCACCAAGAT	356	NC_037332.1
	R: GCCGGATGCAAGCGTAGT		
nanog	F: TGCATTTGCTGGAGACTGAG	107	DQ069776
	R: GTCCCGGTCAAGAAACAAAA		
sox2	F: GAGACGGAGCTGAAGCCGCC	119	NM_001105463
	R: TCTTGGGCCATCTTGCGCCG		
oct4	F: ATGACTTGTGTGGAGGGATGG	338	AF022987
	R: GAACACCTTTCCAAAGAGAACC		

#### Differentiation of the ASCs to adipo-, osteo-, and chondrocytes

The medium of the ASC culture after achieving 50% confluency was replaced with a special medium for the differentiation of the cells into adipocytes, osteocytes, and chondrocytes according to the protocols (A1007001, A1007201, A1007101, Thermofisher) containing antibiotic–antimycotic solution and 1% of BSA. The media were replaced every two days during the 14 days of culture. The control cells were cultured in the same media with antibiotic–antimycotic solution and 1% of BSA for the same period. The culture was divided for staining with appropriate markers of differentiation (the preliminary experiment) and for evaluation of the mRNA expression (experiment 2).

The differentiated adipocytes were washed three times with distilled water, incubated with the Oil Red O working solution composed of 0.30 g of Oil Red-O (189400250, Thermofisher) and 100 ml of 2-propanol for 15 minutes at room temperature, observed with an inverted microscope (Olympus IX70), and visualized.

Prior to Alizarin Red staining, the differentiated osteocytes were washed in PBS and fixed in 95% methanol for 10 min. Alizarin Red S (042040.06, Thermofisher) solution at 2% was added for 5 min; then, the osteocytes were rinsed with distilled water and imaged under an epifluorescence inverted microscope to visualize the Ca^2+^ deposits.

To confirm the differentiation of the cells into chondrocytes, the cells were washed in PBS and stained with 0.1% Safranin-O solution prepared in 20% ethanol (1.59010, Wilmington, DE) for 30 min. The cells were then washed with 95% ethanol and evaluated under a light inverted microscope. The presence of blue-stained clusters of cells was evidence of a positive reaction.

### Experiment 1. Retinoid acid’s effect on metalloproteinase and TIMP mRNA expression and content in ASCs

For the ASC culture, after achieving 70% confluency, the medium was replaced with DMEM containing 1% BSA and antibiotic–antimycotic solution, and the cells were stimulated for 24 h with RA (100 nM, R2625, Sigma). The dose was selected in the preliminary experiment based on not being disturbed by the RA, the cell viability, proliferation, cytotoxicity (MTT-1, 11465007001, Merck, data not included), and the available literature. In the cells, the mRNA expression for MMPs: 1–3 and TIMPs: 1–3 was determined by real-time PCR, whereas in the cells after extraction and in media, the concentration of MMPs: 1–3 and TIMPs: 1–3 was evaluated by ELISA.

### Experiment 2. Metalloproteinase and TIMP mRNA expression in adipocytes, osteocytes, and chondrocytes differentiated from ASCs

After differentiation from ASCs, the adipocytes, osteocytes and chondrocytes were examined by real-time PCR to determine the mRNA expression for MMPs: 1–3 and TIMPs: 1–3 was determined by real-time PCR.

### Determinations

#### Total RNA isolation and reverse transcription

Antler stem cells and differentiated osteoblasts, chondrocytes, and adipocytes were sonicated; then, the total RNA was isolated with TRI-Reagent, according to the manufacturer’s instructions. After extraction, the purity and RNA concentration were assessed with a NanoDrop 1000 spectrophotometer (ThermoFisher). The wavelength ratio for all samples neared 2.0 for 260/280 nm and ranged between 1.8 and 2.2 for 260/230 nm. Then, 1 μg of RNA was calculated based on the spectrophotometric measurement and reverse-transcribed using the High-Capacity cDNA Reverse Transcription Kit (4368813, Applied Biosystems, Cheshire, UK), which contained a MultiScribe™ reverse transcriptase, random primers, RNase Inhibitor, MgCl_2,_ dNTP mixture, and Nuclease-free H_2_O. The samples were incubated at 25°C for 10 min followed by 37°C for 2 h. Finally, to inactivate the reverse transcriptase, the temperature was increased to 85°C for 5 min. The obtained cDNA was kept at -20°C until further analysis.

#### Real-time PCR

The mRNA expression of OCT4, NANOG, SOX2, MMPs (1–3), and TIMPs (1–3) in the ASCs and MMPs: 1–3 and TIMPs: 1–3 in the differentiated osteocytes, chondrocytes, and adipocytes was analyzed by real-time PCR using an Applied Biosystems Real-Time 7900 system (Applied Biosystems), with SensiFAST SYBR Hi-ROX Kit (BIO-92002, Bioline Reagents, London, UK), according to the manufacturer’s instructions. Gene-specific primer sequences were designed using Primer Express Software v.3. The final PCR mix (10 μl) contained 3 μl of reverse-transcribed cDNA (15 ng), 5 μL of SensiFAST SYBR Hi-ROX Mix (SYBR Green and 3 Mm of MgCl_2_), and 0.2 μl of the forward and reverse primers (with 0.5 μM concentration). Each run was performed in duplicate, and the average was considered as a single sample. The results were normalized according to the best reference gene, glyceraldehyde 3-phosphate dehydrogenase (GAPDH), and the two other genes (β-ACTIN, 18S ribosomal RNA) chosen by the NormFinder software (Aarhus University, Denmark). The primer sequences are presented in Table 1. For efficiency evaluation, standard curves consisting of serial dilutions of the cDNA were plotted, and the best cDNA concentration was chosen for further analysis. The first stage of the reaction was the initial denaturation of the strand and activation of the polymerase (95°C for 2 min). The next stage consisted of 45 cycles of successive reactions: denaturation (95°C for 5 sec), primer annealing, and elongation of PCR products (6°C for 20 sec). To ensure the reaction’s specificity, the melting curves of the PCR products were analyzed after the amplification was completed. The data obtained were analyzed using the Miner program. Control reactions lacking the template or primers were performed to confirm that products were free of primer-dimers and genomic DNA contamination, respectively. The PCR products were sequenced and compared with the appropriate genes in the NCBI.

#### ELISA

Concentrations of MMPs: 1–3 and TIMPs: 1–3 after validation in cell lysates and culture media were evaluated using ELISA, according to the procedure included in the kits. All kits were predicted originally for humans and purchased from ThermoFisher. The MMP-1 (EHMMP1) assay’s sensitivity was 8 pg/ml. The MMP-2 (KHC3081) assay’s sensitivity was 1 ng/ml. The mean intra- and inter-assay CVs were 3,3% and 5,6%, respectively. The MMP-3 (BMS2014-3) assay’s sensitivity was 0,5 pg/ml. The mean intra- and inter-assay CVs were 6,1% and 4%, respectively. The TIMP-1 (EH456RB) assay’s sensitivity was 40 pg/ml. The TIMP-2 (EHTIMP2) assay’s sensitivity was 2 pg/ml. The TIMP-3 (EH458RB) assay’s sensitivity was 0,05 ng/ml. The mean intra- and inter-assay CVs for MMP-1, TIMP-1, TIMP-2, and TIMP-3 were 10% and 12%, respectively. Each run was performed in duplicate, and the average was considered as a single sample and detected at 450 nm.

### Statistical analysis

GraphPad PRISM (Version 9.4.1, San Diego, CA, USA) was used for data analysis. Analyses of mRNA expression in the preliminary experiment and experiment 2 were evaluated using one-way ANOVA, followed by Tukey’s test; the mRNA expression and concentration were determined between different MMPs and TIMPs and the control versus RA stimulation in experiment 1, using two-way ANOVA. All the numerical data are expressed as the arithmetic mean ± standard error of mean (SEM). The statistical significance was P *<* 0.05.

## Results

### Preliminary experiments

#### Determination of the pluripotent markers in mRNA expression in ASCs

**Fig 1:**
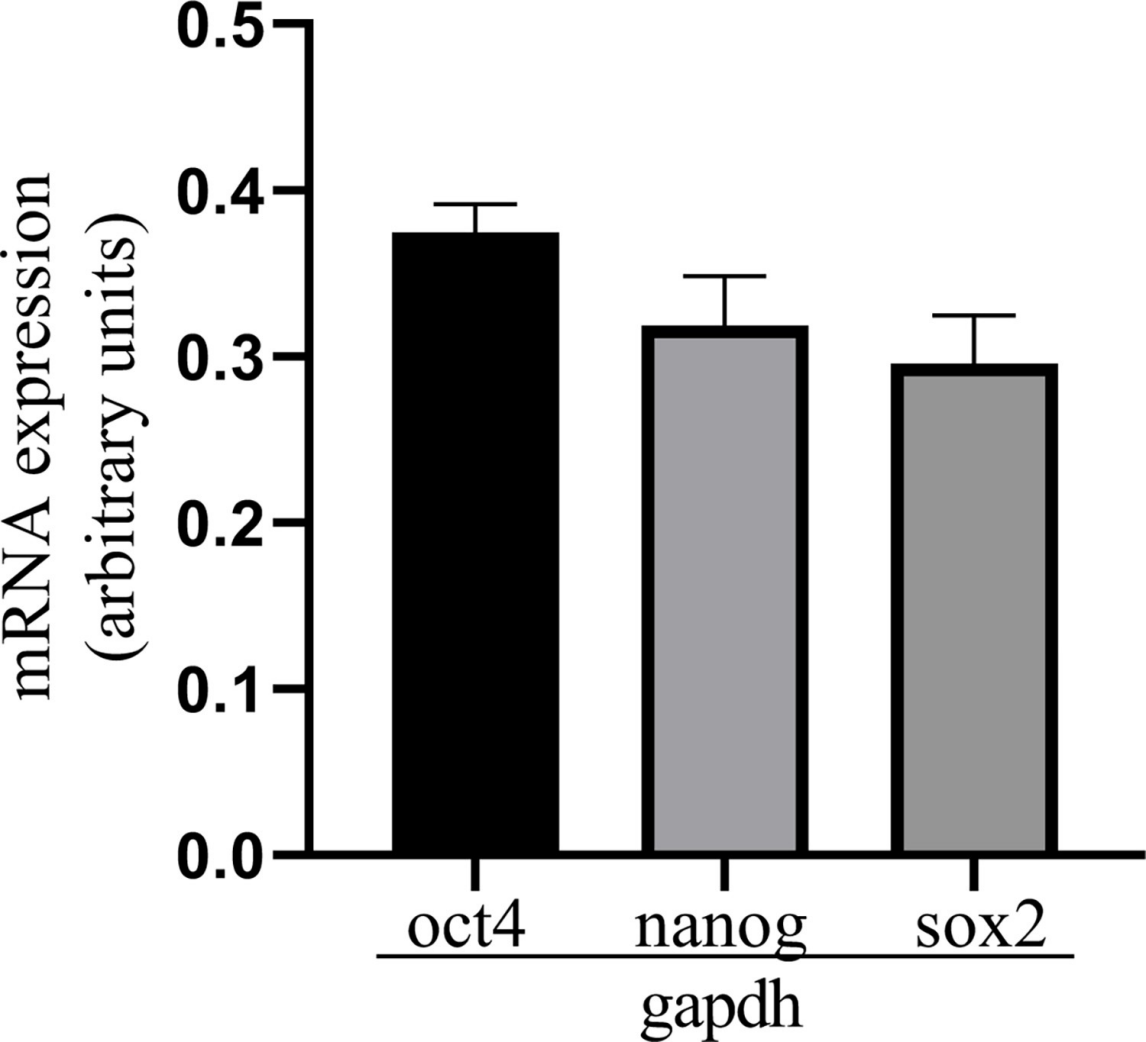
mRNA expression of OCT4, NANOG and SOX2 in antler stem cells. Data were normalized against GAPDH for mRNA expression using GraphPad PRISM. Each bar represents one experimental group with SEM. Statistical differences were analysed by one-way ANOVA followed by the Tukey’s post hoc test.

The mRNA expression of OCT4, NANOG, and SOX2 was identified on a similar level in antler cells, which shows the pluripotency of these cells (P > 0.05; Fig 1). There were no statistical differences between the cells isolated from the apical and pedicle antler layers (P > 0.05).

#### Differentiation of the ASCs to adipo-, osteo-, and chondrocytes

**Fig 2:**
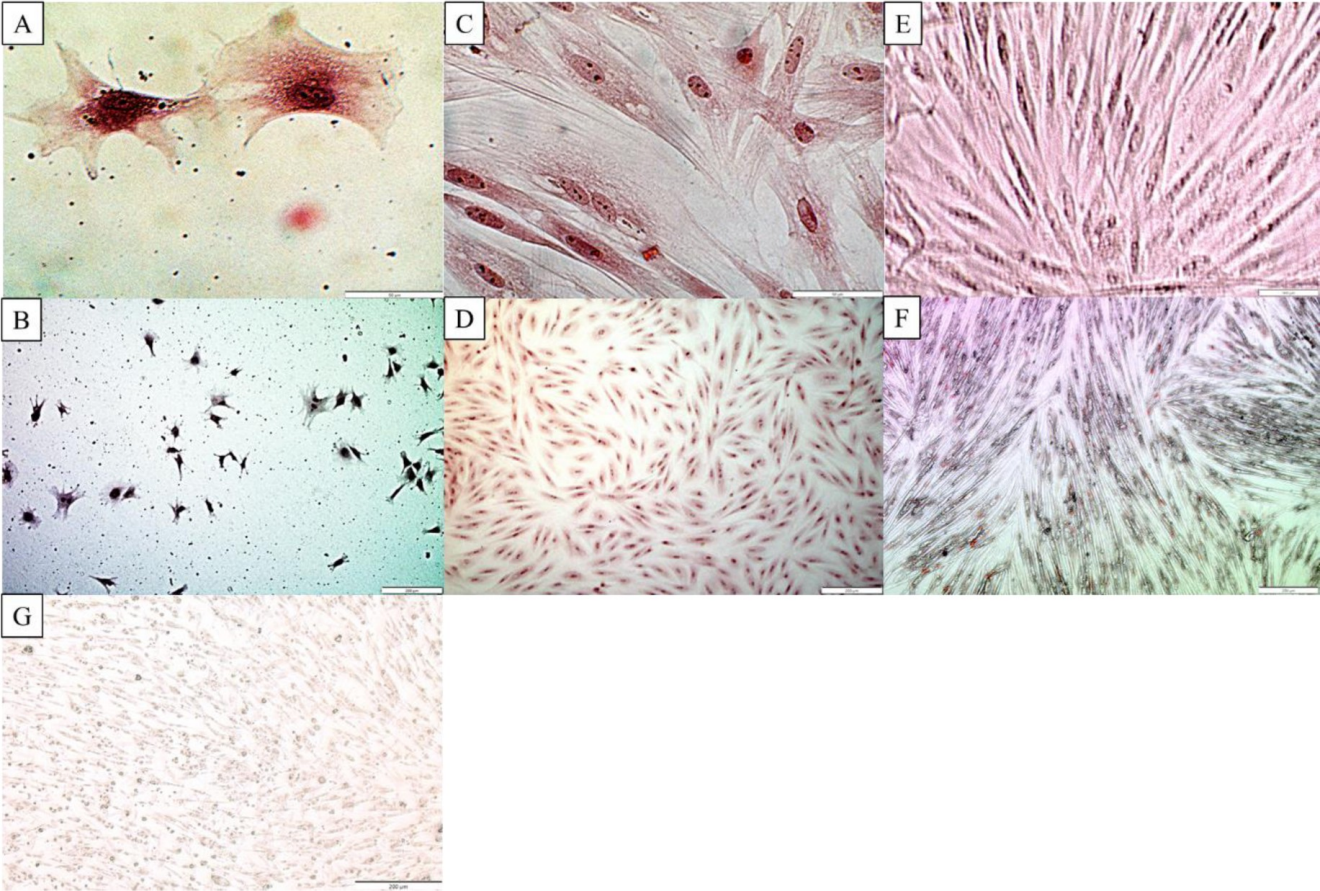
Antler stem cells after differentiation to osteocytes (A, B, staining with Alizarin Red), chondrocytes (C, D, staining with safranin-O) and adipocytes (E, F, staining with Oil Red O). Negative control represented antler stem cells cultured 14 days without differentiation (G).

The staining of ASCs with Alizarin red was positive and identified calcium-containing differentiated osteocytes (Fig 2A and 2B), Safranin-O made visible the glycosaminoglycans in the differentiated chondrocytes (Fig 2C and 2D), and Oil Red O was positive and showed red lipid droplets of differentiated adipocytes (Fig 2E and 2F). The negative control subjected to differentiation to osteocytes, adipocytes, and chondrocytes in the same way as the ASCs did not reveal positive staining after two weeks of culture (Fig 2G).

### Experiment 1. Retinoid acid’s effect on metalloproteinase and TIMP mRNA expression and content in ASCs

**Fig 3:**
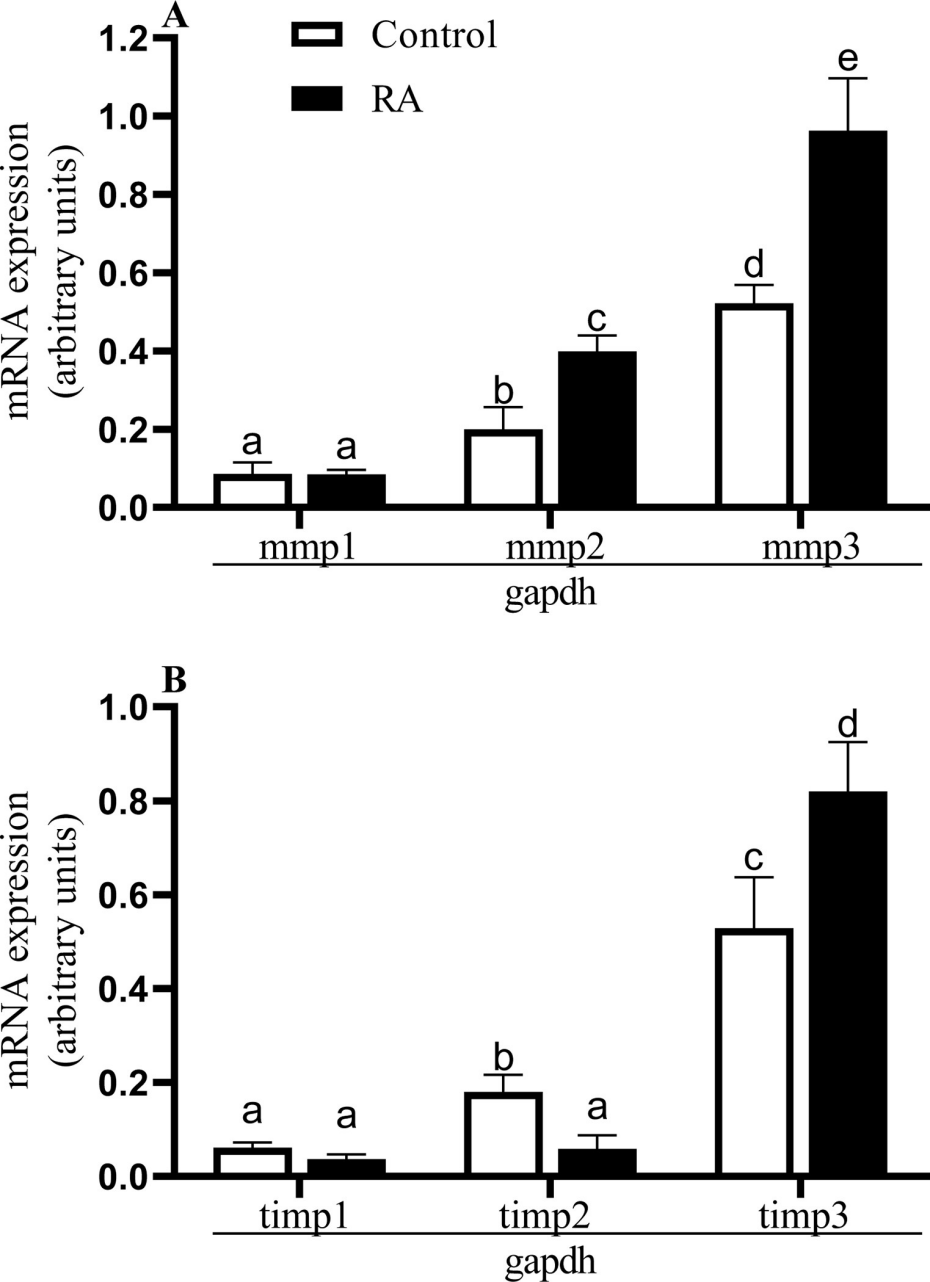
mRNA expression of MMPs: 1–3 (A) and TIMPs: 1–3 (B) after stimulation with retinoic acid (RA) in antler stem cells. Data were normalized against GAPDH for mRNA expression using GraphPad PRISM. Each bar represents one experimental group with SEM. Statistical differences were analysed by one-way ANOVA followed by the Tukey’s post hoc test. Data were normalized against GAPDH for mRNA expression using GraphPad PRISM. Each bar represents one experimental group with SEM. Statistical differences were analysed by one-way ANOVA followed by the Tukey’s post hoc test. The lowest statistical significance was P < 0.05. Different letters indicate statistical differences (P < 0.05) between each experimental pair (control versus RA stimulation).

Retinoid acid stimulated the mRNA expression of MMP-2, MMP-3, and TIMP-3 and inhibited TIMP-2 in the ASCs (P < 0.05), whereas the mRNA expression for MMP-1 and TIMP-1 after RA was not changed (P > 0.05; Fig 3A and 3B).

**Fig 4:**
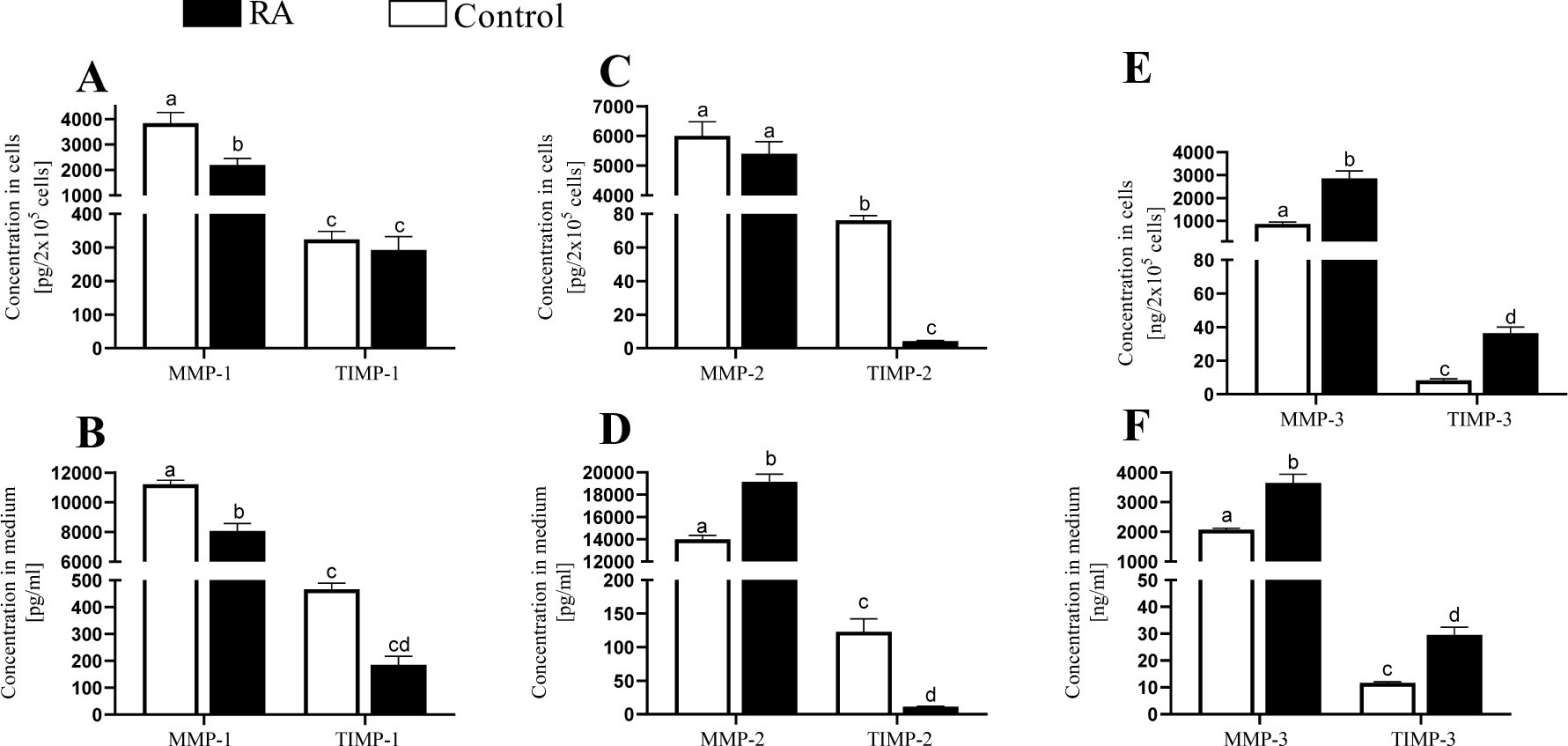
Output of MMPs: 1–3 (A) and TIMPs: 1–3 (B) after stimulation with retinoic acid (RA) in antler stem cells. Concentration in the cells is presented on graphs A, C and E and concentration in the medium on graphs B, D and F. Each bar represents one experimental group with SEM. Statistical differences using two-way ANOVA were analysed between different MMPs and TIMPs and control versus RA stimulation. The lowest statistical significance was P < 0.05. Different letters indicate statistical differences (P < 0.05) between each experimental pair (control versus RA stimulation).

The content of the MMPs and TIMPs was evaluated as the concentration of these proteins in ASCs and the medium. The MMP-1 concentration in the ASCs and medium was inhibited under the influence of RA (P < 0.05), whereas the TIMP-1 concentration was not changed (P > 0.05; Fig 4A and 4B). The MMP-2 concentration was higher only in the medium stimulated with RA (P < 0.05; Fig 4D). The TIMP-2 output was lower both in the ASCs and the medium after RA treatment (P < 0.05; Fig 4C and 4D). Retinoic acid increased MMP-3 and TIMP-3 output both in the ASC and in the medium (P < 0.05; Fig 4E and 4F).

### Experiment 2. The profile of MMP and TIMP mRNA expression in adipocytes, osteocytes, and chondrocytes differed from that in the ASCs

**Fig 5:**
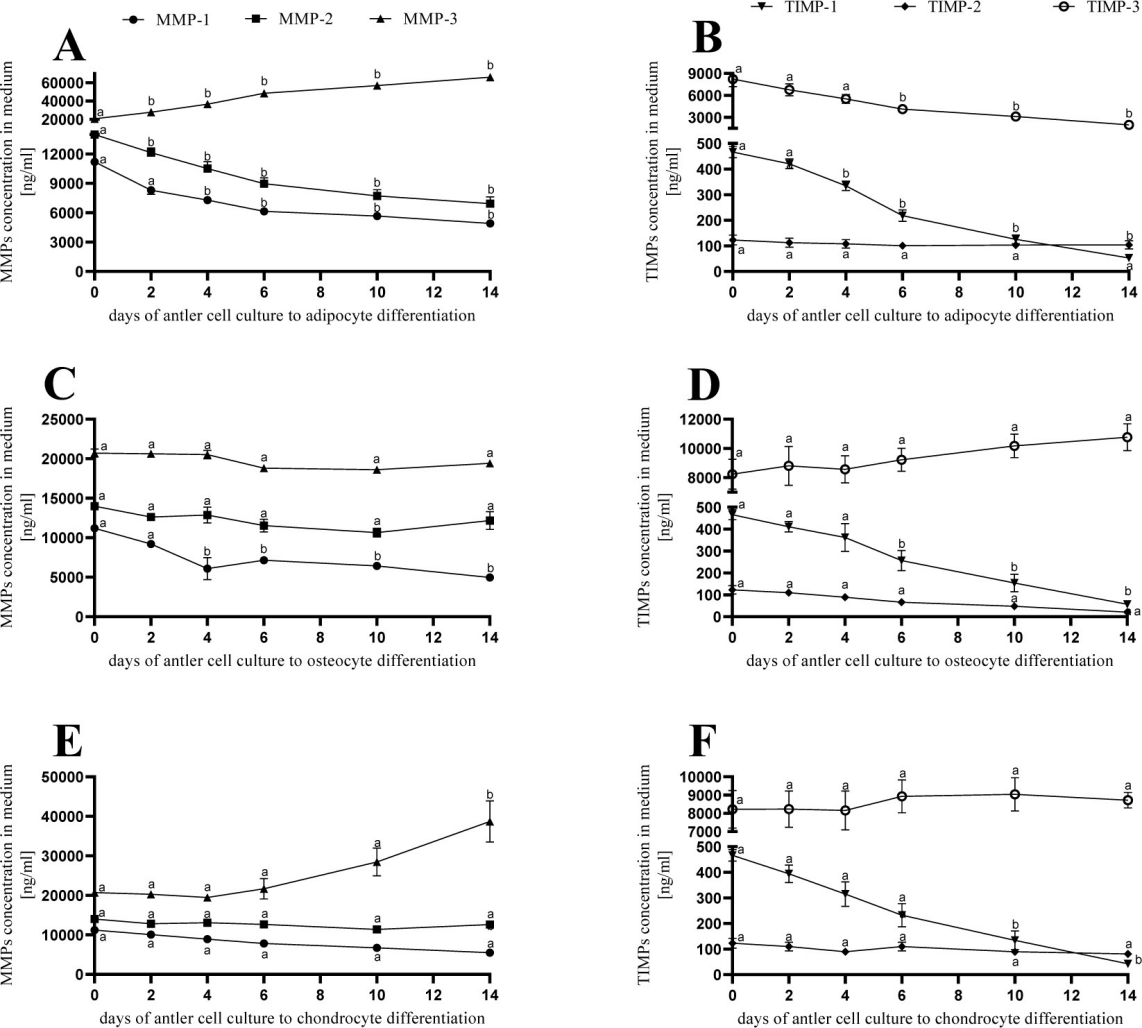
Profile of MMPs: 1–3 (A, C, E) and TIMPs: 1–3 (B, D, F) concentration during differentiation of antler stem cells into adipocytes, osteocytes and chondrocytes. Each bar represents one experimental group with SEM. Statistical differences were analysed by two-way ANOVA of variance followed by the Tukey’s post hoc test. The lowest statistical significance was P < 0.05. Different letters indicate statistical differences between different days of differentiation for each MMP or TIMP (P < 0.05).

Downregulation was observed for the MMP-1 mRNA expression during adipocyte and osteocyte differentiation (Fig 5A and 5C; P < 0.05) and for the MMP-2 mRNA expression during adipocyte differentiation (Fig 5A; P < 0.05), as well as upregulation for the MMP-3 mRNA expression during adipocyte and chondrocyte differentiation (Fig 5A and 5E; P < 0.05). The stable profile of mRNA expression during differentiation was noticed for MMP-1 during chondrocyte differentiation (Fig 5E), for MMP-2 during osteocyte and chondrocyte differentiation (Fig 5C and 5E), and for MMP-3 during osteocyte differentiation (Fig 5C; P > 0.05).

Downregulation was observed for the TIMP-1 mRNA expression during adipocyte, osteocyte, and chondrocyte differentiation (Fig 5B, 5D and 5F) and for TIMP-3 mRNA expression during adipocyte differentiation (Fig 5B; P < 0.05). The stable profile of mRNA expression during differentiation was noticed for the TIMP-2 during adipocyte, osteocyte, and chondrocyte differentiation (Fig 5B, 5D and 5F) and for TIMP-3 during osteocyte and chondrocyte differentiation (Fig 5D and 5F; P > 0.05).

## Discussion

This report is the first where MMP action in ASCs was examined. We evaluated both MMPs: 1–3 and TIMPs: 1–3 mRNA expression and content in the ASCs and in the medium for the comprehensive effect of these proteases’ action. We showed the profile of the mRNA transcripts for the MMPs and TIMPs in differentiated osteocytes, chondrocytes, and adipocytes from the ASCs. Moreover, we proved that RA alters MMP and TIMP action in red deer ASCs.

Both the location of the cells obtained from the antler as well as the stage of its growth influenced the pluripotency potential. In our case, it was tissue collected 40 days after antler casting from the pedicle periosteum layer according to Wang et al. [10]. Seo et al. [31] isolated antler stem cells *in vivo* after anesthesia via biopsy approximately 45–60 days after antler growth initiation. There was effective differentiation of the ASCs to osteocytes, chondrocytes, and adipocytes, and the mRNA expression for OCT4, NANOG and SOX2 proved the pluripotency of the ASCs. This result confirmed the proper choice of the antler growth stage and the antler tissue layer (pedicle) for the experiments.

The ability of the cells to differentiate into cells with more specialized functions and more restricted fate is fundamental to the development of multicellular organisms but also for regenerative processes, as well as the daily maintenance of every tissue in the body. The decision of a precursor cell to differentiate is strongly context-dependent and may be influenced by changes in the surrounding microenvironment. MMPs are proteolytic mediators of extracellular matrix remodeling and thereby may provide the necessary changes to the microenvironment triggering cellular differentiation during developmental processes. To date, the expression of MMPs has been demonstrated both repeatedly in stem cells [32] and in tumour cells [33]. In cancer cells, increased expression of MMPs affected the proliferation during development of the tumour microenvironment and altered the bioavailability of growth-regulating factors [34]. Although the expression of MMPs has been proven in stem cells, the concentration has so far not often been determined either in cells or in the medium after culture. Our results indicated that mRNA expression was the highest for MMP-3 in the ASCs. Meanwhile, the highest concentration as a sum of the concentrations from cells and the medium was for MMP-2. Such results indicate that some posttranscriptional processes occurred in these cells. Among the three types of TIMPs, TIMP-3 showed the highest expression and concentration in the ASCs. This type of inhibitor, possibly by forming a complex with MMP-3, causes an attenuation of the action of the functional protease (MMP-3) in the ASCs in favor of MMP-2. In addition, the concentration for all types of MMPs was higher than for the TIMPs, which in turn suggests the high activity of all MMPs in ASCs. Generally, we are convinced that the different concentration of MMPs and TIMPs inside ASCs and in the medium was received because in the medium occurred the accumulation of proteins which were metabolized during each two days of cell culture (the medium was replaced every other day during the differentiation process). Whereas the concentration in cell pellet was much lower than in the medium because it reflected the current state of protein action.

Expression of the MMPs and their inhibitors were differently regulated after ASC differentiation to osteocytes, chondrocytes, and adipocytes; mostly, there was downregulation of mRNA expression. MMPs play an important role in bone resorption and matrix degradation [35]. MMP-1 makes endogenous collagens more susceptible to destruction by other MMPs [36], and MMP-2 degrades partially denatured collagens and gelatin [37]. Knockdown of MMP-1 resulted in an increase in osteogenic marker genes, while overexpression of MMP-1 reversed this effect [38]. We found a stable profile of mRNA expression for MMP-2 and 3, TIMP-2 and -3, and a slight decline for the MMP-1 and TIMP-1 and -2 profiles during the differentiation of ASCs to osteocytes, which predispose these cells to future therapy in bone disorders. During chondrocyte differentiation, we observed an increase only in mRNA expression for MMP-3 but a decrease for TIMP-1. Cartilage degradation is mediated by MMPs [39], and MMP-3 is particularly linked to pathological conditions in cartilage [40]. A decrease in the mRNA expression for MMP-3 was shown after the differentiation of adipose-derived mesenchymal stem cells into chondrocytes-like cells under the influence of insulin growth-like factor 1 and bone morphogenic protein 2 [40]. We found an increase in the mRNA expression profile for MMP-3 during ASC differentiation to adipocytes and a decrease in the MMP-2 mRNA expression. Moreover, we observed downregulation of the mRNA expression during differentiation to adipocytes for MMP-1 and TIMP-1 and -3.

The action of MMPs is mediated by different factors dependent on the character of the cellular process, and one such potential mediator is RA. After stimulation of the ASCs with RA we found upregulation of MMP-2 and -3 mRNA expression and output, in contrast to the MMP-1 output. This finding is in agreement with the study of Darmanin et al. [41] on murine dendritic cells and the study of Pourjafar et al. [27] on rat bone marrow-derived mesenchymal stem cells, where the cells treated with RA showed enhanced MMP-2 production. In another study, MMP-2 activity and mRNA expression were suppressed by RA in human fetal palate mesenchymal cells [42]. This inconsistency is due to the different roles of MMPs in different tissues and cells that cause differences in RA signaling. Interestingly, there was an increase in the mRNA expression and content after RA treatment both for MMP-3 and TIMP-3 in ASC, which makes RA necessary for establishing a balance between MMP-3 and TIMP-3. Allen et al. [11] showed that RA regulated the differentiation of ASCs during their intense growth. Deciphering the functional role of retinoids in this unique regeneration model provides the basis for developing effective therapies. Moreover, other factors are involved in RA signaling pathway apart from MMPs and TIMPs, such as transforming growth factor β (TGF). There is complex crosstalk between the TGF and retinoid signaling pathways, where TGF can have opposite effects depending on the physiologic (developmental state, age, organ system, cell type, species) or pathologic setting [34, 43].

In summary, our results clearly demonstrated the different production of MMPs and TIMPs in ASCs, where RA played a regulatory function. Moreover, MMPs seem to play a tissue-specific role during differentiation of these cells to adipocytes, osteocytes, and chondrocytes, which may be relevant when ASC is used as an extract for wound healing acceleration or as a cell transplant to the affected tissue. Continued research both on the positive and negative effects of MMPs and TIMPs, particularly in cells differentiated from ASCs, is needed. Our results indicate that some other molecular pathway, not direct inhibition of MMP action through TIMPs are possible during antler stem cells`differentiation, involving other proteinases and factors. Although ASCs seem desirable in wound treatments and have already been used in such practices [30] we plan to expand our research to examine the effects of other potential mediators of differentiation and stem cell proliferation.
